# Supplementary material for: Evaluating “Conmigo, Contigo, Con Todo”: Effects of a community mental health initiative on Afro-Colombian teachers
Source: Glob Ment Health (Camb). 2025 Oct 16;12:e117. doi: 10.1017/gmh.2025.10074 (PMC12641320; doi:10.1017/gmh.2025.10074)
Supplement: Gonzalez-Ballesteros et al. supplementary material [file S2054425125100745sup001.docx]

## Supp Table S2. Detailed CONSORT Allocation of Clusters and Participants

| Stage | Clusters | Participants |
| --- | --- | --- |
| Assessed for Eligibility | 20 | – |
| Randomized | 8 | 32 |
| Allocated to Intervention | 4 | 28 |
| – Received Intervention | 4 | 28 |
| – Did Not Receive | 0 | 0 |
| Allocated to Control | 4 | 4 |
| – Received Intervention | 0 | 0 |
| – Did Not Receive | 4 | 4 |
| Analysed (Intervention) | 4 | 28 |
| Analysed (Control) | 4 | 4 |
| Lost to Follow-up | 0 | 0 |
| Discontinued Intervention | 0 | 0 |
| Excluded from Analysis | 0 | 0 |

Note. This table provides a detailed breakdown of the CONSORT allocation process, reflecting the initial assessment of 20 clusters, randomization into 8 clusters (4 intervention, 4 control), and the final analyzed sample, with no losses or exclusions. The imbalance (n = 28 intervention, n = 4 control) limits between-group comparisons.
